# Research on the Effectiveness of Probabilistic Stochastic Convolution Neural Network Algorithm in Physical Education Teaching Evaluation

**DOI:** 10.1155/2022/4921846

**Published:** 2022-04-27

**Authors:** Wei Cui

**Affiliations:** Physical Education Department, Shanghai University of Finance and Economics, Shanghai 200433, China

## Abstract

In practice, PE teaching evaluation based on probabilistic convolutional neural network still faces some practical problems. At present, the existing research mainly focuses on how to improve the accuracy of PE (physical education) teaching evaluation, but ignores the balance between accuracy and speed of the model, which is the key to achieve efficient PE teaching estimation. Aiming at the problem of optimization contradiction existing in the traditional probabilistic stochastic convolution neural network regression method, a position adaptive probabilistic stochastic convolution neural network regression method was proposed. Firstly, the basic principle of probabilistic and random convolution neural network regression method is given. Secondly, the contradiction and reasons between hot trial regression and coordinated regression are analyzed. It is found that the process heat will return to the optimization with irreconcilable contradiction with the coordinates due to the lack of learning parameters when the hot trial transforms the coordinates. The optimization contradiction will make the model tonot obtain the exact coordinates of the nodes. Then, based on the above analysis, the learnable parameters are introduced into the Softmax function, and the position adaptive Softmax model is proposed. Combining the model with the probabilistic stochastic convolution neural network regression method, the position adaptive probabilistic stochastic convolution neural network integral regression method is obtained. In order to reduce the training cost of this method, a simplified training strategy is proposed. Finally, the simulation software MATLAB is used for verification, and the functions of sample maintenance, probabilistic stochastic convolution neural network training, and neural network evaluation are realized. The experimental data show that the probabilistic stochastic convolutional neural network is feasible for teaching quality evaluation, meets the accuracy requirements, and indeed provides a convenient and practical tool for PE teaching quality evaluation.

## 1. Introduction

In the field of teaching management in colleges and universities, academic performance and classroom performance are the main basis for the analysis of learning effect, so as to complete the evaluation of students' learning ability in PE, professional courses, and public courses. A large number of academic results cause difficulties for teachers' teaching management. With the optimization and update of information processing technology and big data technology, educators place their hopes on intelligent mining algorithm [1] for student score management, and data mining has gradually become an effective tool for subject score management and learning effect analysis in colleges and universities.

The key significance of data mining in college sports lies in which it can take into account students' physical condition, classroom performance, after-school training, and other diversified evaluation indexes to comprehensively evaluate students' PE learning effect, avoiding subjectivity and one-sidedness in the analysis of learning effect [2]. Compared with manual data processing, the efficiency of data mining is greatly improved and the evaluation accuracy is more accurate. On the one hand, the results of data mining can be used as the basis of teaching quality evaluation, and on the other hand, it can provide the decision-making basis for teaching managers to optimize the teaching evaluation model. Neural network is based on the self-learning mathematical model. It can analyze a large number of complex data and complete pattern extraction and trend analysis which are extremely complex for human brain or other computers [3]. A neural network system consists of a series of processing units, called nodes, that are similar to neurons in the human brain. These nodes are connected to each other through the network, and if there is data input, they can perform the work of determining the data pattern [4]. The neural network is composed of the interlinked input layer, the intermediate layer (or the hidden layer), and the output layer. The middle tier consists of multiple nodes that do most of the network work. The output layer outputs the execution results of data analysis. The basic task of intelligent sports is to obtain the movement changes of the human body during exercise through equipment, so as to achieve the analysis and guidance of the standard degree of exercise, which is divided into professional athletic field and civil fitness field [5]. The traditional way is mechanical, acoustic, electromagnetic, optical, and so on. Mechanical use of external sensors and rigid support can affect limb movement. The disadvantages of acoustic type and electromagnetic type are easy to be interfered by external environment and large time delay. Optical results are more accurate, but they are expensive and take a long time to process. In recent years, new technology is constantly emerging, based on accelerometer and gyroscope, magnetometer inertial measurement technology, but the technology of professional equipment dependence is stronger, so it will affect athletic performance and its expensive [6, 7] more than the traditional methods used in the professional training of competitive sports; the training demand is high, needs to spend a lot of human resources, and realizes intelligent training of professional sports.

With the development of new computer technologies such as artificial intelligence and big data technology in recent years, the application of physical education evaluation and guidance has been deepening. The data acquisition and analysis system based on Intelligent bracelet wearing equipment can measure and intelligently analyze the movement track of students by collecting the data of students' bracelets [8], capture the movement records of students in the process of sports training, and then further analyze the data and observe whether the actions meet the specifications [9]. There is video-based analysis of human movements. For example, Kinect somatosensory equipment is used to capture human body joints, which is used for supplementary training of learners in golf action training system [10, 11]. There is a sports action teaching platform with more immersive experience based on virtual reality technology, which simulates human movement scenes to achieve realistic action simulation effects [12]. With the deepening of deep neural network research and the rapid development of hardware, artificial intelligence technology based on deep learning performs well in the field of effectiveness identification of physical education teaching evaluation [13]. This kind of method can accurately predict the data information of human bone points on every frame of video and combine them into human skeleton. It provides accurate basic data for motion recognition and posture matching based on the validity characteristic information of video physical education evaluation and can realize accurate analysis of the validity of physical education evaluation in time and space. It has the advantages of strong practicability and wide application range. The method based on dynamic Bayesian network (DBN) expands the Bayesian network in the time dimension [14] and then decomparts the complex motion into simple variables. This method can learn the probability dependence relationship between variables and the rule of their changes with time. In the process of dynamic identification, DBN can design the corresponding network structure for specific problems and can fuse all kinds of information for reasoning. At the same time, in order to optimize the configuration of information, it can also set different weights for different information according to its source and confidence degree. DBN is introduced into behavior recognition for attitude matching [15], pointing out that because it contains more hidden nodes and observation points, DBN can provide more object description details compared with HMM. A primitive is proposed to solve the pose matching in behavior recognition of a specific topic, where the primitive is composed of features describing context information [16]. DBN can integrate different weak information features and strengthen them, thus improving the efficiency and robustness of matching. Hierarchical Bayesian network was used to match pairs' behaviors, and DNN was used to evaluate multipart physical education teaching [17]. The training of DBN is simpler than HMM, but the design is much more complex than HMM. In addition, due to the need for a large number of characteristic parameters, the method of dynamic Bayesian network has a large amount of calculation and high complexity. The data collection of college students' sports information has significant information characteristics. Compared with the statistics of students' scores by office software in traditional teaching, big data technology pays more attention to the integration of data from teaching sensors, teaching management platforms, and other channels. The data types required to analyze the physical education learning effect of college students mainly include students' basic information, physical education training, after-class physical education training, sports assessment results, and sports competition results [18].

Therefore, using the advantages of data mining in the field of association analysis, this study proposes a method for analyzing the learning effect of college students' physical education based on probabilistic stochastic convolution network algorithm. In view of the existing problems in PE teaching estimation based on convolutional neural network, combined with the actual requirements of PE teaching estimation (reduce model computation, improve model reasoning efficiency, and ensure model accuracy), this study focuses on the network architecture, training strategy, and preprocessing and postprocessing of probabilistic stochastic convolutional neural network in the physical education teaching estimation method. Through the attitude matching algorithm proposed in this study, the attitude matching and action matching are realized to evaluate the standard degree and give the score, identify the deviant limbs in the nonstandard movements, guide students to learn and exercise the basic movements in physical education, and provide the realization method for the examination and evaluation of the basic movements in physical education. This study realizes the assessment and evaluation function of basic movement teaching in intelligent physical education teaching, solves the problems of obtaining teachers' guidance in learning basic movement of physical education in extracurricular physical exercise scenes and the workload of teachers' checking exercise situation, and contributes to the teaching and evaluation of basic movement of physical education.

## 2. Physical Education Teaching Evaluation Based on Probabilistic Stochastic Convolution Neural Network

### 2.1. Probabilistic Convolutional Neural Network Evaluation Method

**Figure 1:**
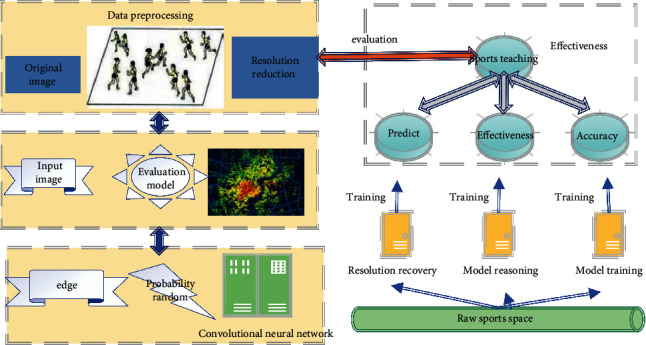
Process of the PE teaching evaluation method based on probabilistic stochastic convolutional neural network.

Sports management data mining platform can share students' basic information with teaching platform through special data interface and import relevant data into data mining platform. Students' training in class is generally recorded by teachers in class and on the teaching management platform, and sports competition results are summarized and recorded on the information platform. Through the “independent sports training monitoring system,” we can collect students' extracurricular training situation, analysis, and processing and then sent to the teaching information management platform of the background database, as the basis for analyzing students' sports learning effect. Therefore, two key big data acquisition technologies need to be introduced here: one is the teaching information management platform and the other is an independent sports training monitoring system. The PE teaching evaluation process of probabilistic stochastic convolutional neural network is shown in Figure 1.

Big data preprocessing generally includes data cleaning, integration, transformation, and other steps, aiming to reduce redundant noise information in data, supplement missing data, and delete repetitive data. In order to improve the accuracy of the analysis of college students' PE learning effect and reduce the difficulty of data analysis, a unified format is adopted to express the students' PE learning effect, and a more accurate PE learning effect analysis conclusion can be obtained based on real and reliable data.

Importing heterogeneous data from teaching information management platform, it is necessary to represent the data in a standardized form. For example, the expression forms of students' performance *y* in the 100-meter race are different, so the normalization operation is carried out here:

(1) $$\begin{array}{c} {\hat{y}}_{i}=\frac{\left(y_{i}-y_{\min}\right)}{\left(y_{\max}-y_{\min}\right)}. \end{array}$$

In addition, it is necessary to delete invalid personal scores, invalid class scores, information of students who have changed majors, information of physical education students, etc., and then supplement the missing data based on the machine learning method. The method is divided into two steps. First, the correlation analysis of the students' initial sports data is carried out to obtain the known value attribute data with the greatest correlation with the missing data. Secondly, a data prediction model is constructed based on neural network algorithm to estimate the missing part of students' PE scores. The prediction error of the method is small, and the physical characteristics and training results of students in the conventional environment are considered scientifically. It is a good method to solve the problem of missing data of students' PE results.

### 2.2. Probabilistic Position Adaptive Convolutional Neural Network Softmax Model for PE Teaching Evaluation

The position adaptive Softmax model can parameterize the probabilistic stochastic convolution neural network method, and the position adaptive weight function can eliminate the contradiction between thermal diagram regression and coordinate regression. In addition, it is necessary to parameterize the probabilistic *p* stochastic convolution neural network method, which can not only solve the optimization contradiction *R* between the thermogram regression and coordinate regression but also fully enjoy the advantages of the thermogram regression method:

(2) $$\begin{array}{c} \xi \left(p,\beta ,m\right)=\frac{R\left(p;\beta ,m\left(p\right)\right)}{\sum R\left(p;\beta ,m\left(p\right)\right)}. \end{array}$$

The first step is to pretrain the thermogram regression model until it converges.

In the second step, the parameters of the thermogram regression model are frozen, and the coordinates of the thermogram predicted by the thermogram regression model and the key points in the training set are used as training samples for the training position adaptive probabilistic stochastic convolutional neural network model.

**Figure 2:**
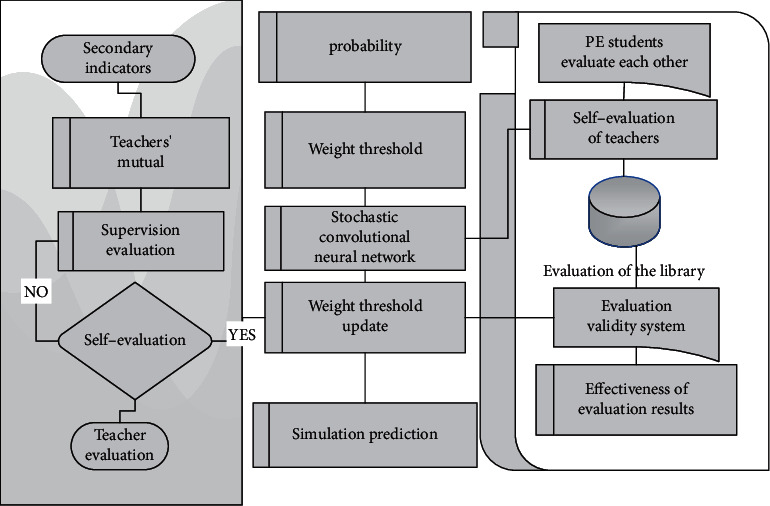
Structural framework of probabilistic stochastic convolutional neural network quality evaluation system for effectiveness of physical education teaching evaluation.

The simplified training strategy can freeze the pretrained thermogram regression model and avoid the interference to the thermogram regression model during the training of the position adaptive probabilistic stochastic convolutional neural network model. This strategy can not only reduce the training costs but also can make position adaptive probability and stochastic convolution neural network method with the existing training good heat to a good combination of the regression model, to avoid the heat to the regression model of training and enhance the position adaptive probability and stochastic generality of the convolution neural network method. It should be noted that the simplified training strategy cannot be applied to the traditional probabilistic stochastic convolutional neural network method because there are no learnable parameters in the process of thermal diagram transformation into coordinates in the traditional method, and no training is required in the second stage. The system structure block diagram is shown in Figure 2.

### 2.3. Effectiveness of Probabilistic Stochastic Convolutional Neural Network in Physical Education Teaching Evaluation

Before the neural network evaluation model is set up, first of all, we should make sure of the evaluation index system; based on the scientific, comprehensive, accuracy, and operability principles, the evaluation index *x* system of higher vocational colleges, for example, the student neural network evaluation index design, is as shown in Table 1; the index system determines the network structure of the teaching quality evaluation model.

The specific steps of establishing the neural network model of student evaluation are as follows.

#### 2.3.1. Determination of Input Layer Node

The teaching evaluation index of students is divided into 4 first-level indicators, 12 second-level indicators, and 12 second-level indicators as the input of the input layer of the neural network. Therefore, the number of nodes in the input layer of the probabilistic stochastic convolutional neural network is correspondingly determined to be 12.

#### 2.3.2. Determination of Node Number of the Output Layer

Since there is only one result of students' teaching evaluation, the output layer of the network is only set as one output node. The value ranges from 0 to 1.

#### 2.3.3. Determination of the Number of Hidden Layer Nodes

So far, how to select the optimal number of hidden layer nodes is still an urgent problem to be solved. Theoretically speaking, if the number of hidden layer nodes selected is too small, the convergence speed of the whole neural network will be slow and difficult to converge. On the contrary, if the number of hidden layer nodes selected is too large, the topology of the neural network will be complicated, the training time of the network will increase sharply, and the error may not be the best. At present, a common method to determine the optimal number of hidden layer nodes is the trial-and-error method. In the trial-and-error method, some empirical formulas can be used to determine the number of hidden layer nodes *x*:

(3) $$\begin{array}{c} y=\sqrt{x+l}+\alpha ,\\ y=\mathrm{lg}x. \end{array}$$

#### 2.3.4. Selection of Activation Function

For the activation function on the hidden layer element, select the tansig hyperbolic tangent function. In the training data sample set, the expected output values of the evaluation results all fall within the interval [0, 1] after normalized processing. Therefore, the activation functions on the output layer units are Sigmoid functions in the form of

(4) $$\begin{array}{c} f\left(x\right)=\frac{1}{1+\sum e^{x}}. \end{array}$$

#### 2.3.5. Establishment of the Model

The output of hidden layer node *h* and output layer node *s* are

(5) $$\begin{array}{c} y_{h}=f\sum_{i}w_{ih}x_{i}+\alpha_{k},\\ o_{s}=g\sum_{i}w_{si}x_{s}+\alpha . \end{array}$$

#### 2.3.6. Initial Setting of Weights and Thresholds

For nonlinear systems, probability, and stochastic convolution neural network connection weights and threshold of the initial value, whether in the process of online learning network will converge to local minimum and whether there is a big relationship, an important requirement is that hope initial weights to make the state of each neuron in the input sum value close to zero, which can ensure the beginning does not fall on the flat area. The weights are usually random and small so that each neuron starts at the place where its conversion function is greatest.

**Figure 3:**
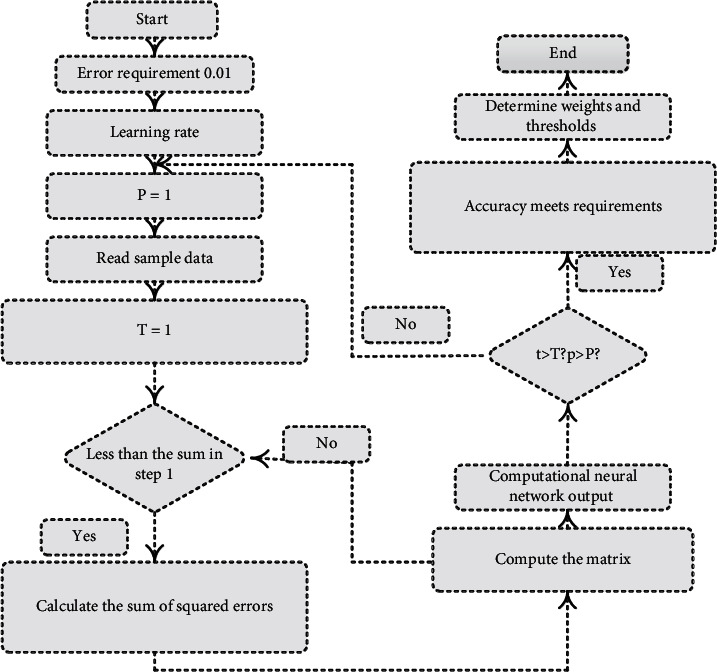
Flowchart of PE teaching evaluation based on probabilistic stochastic convolutional neural network.

The above calculation process can be described in Figure 3.

In the sports action in the teaching process, a sports action completion is a continuous process; every movement process is a video frame frame sequence, and different groups, such as teachers and students, are different. As a result, the length of the action frame sequence to be matched must not be the same as that of the template sequence. In addition, even if the speed is consistent in the process of the action in the ideal state, it is usually impossible to guarantee the same time of the “attention” action at the beginning and end of the action. This requires an algorithm that can overcome the matching problem of different sequence lengths and solve the problem of prolonged or shortened action time or action sequence translation.

**Figure 4:**
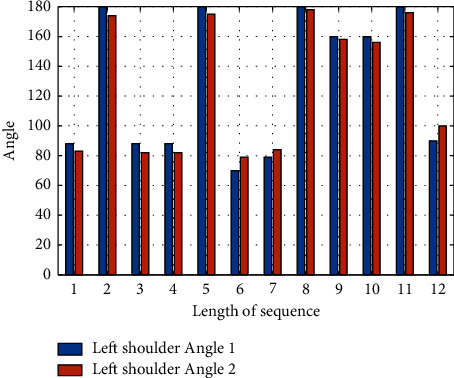
Comparison of shoulder angle changes in different human side movements.

In the process of learning an action, different proficiency of the same person and different people performing the same action will lead to inconsistent sequence length. For example, when the same person performs the same action at the same standard and different speed, the angle change of right elbow is shown in Figure 4.

By Figure 4, we can see that, in different speeds to do the same action, in addition to the differences in time dimension, from the angle of your right elbow body reaction, spatial dimension changes trend is basically the same; in the process of action evaluation, pay more attention to degree of standard of gestures; it is important to accurately reflect in the degree of match body space form. You need to eliminate nuances in speed of action, so you need to look at matching on two sequences of different lengths.

### 2.4. Experimental Design

In order to verify the analysis effect of the optimized probabilistic stochastic convolutional neural network algorithm on student physical education, two classes of students of a major of grade 2018 were taken as objects to conduct an analysis test on the learning effect of college students' physical education. Among them, the knowledge base is the sports information of students, and the sample information is from the campus teaching information management platform, that is, students' basic information, physical education training, physical training after class, physical examination results, and sports competition results. The evaluation library is the setting of evaluation standards for students' learning effects, which are described by poor, general, good, and excellent. After data preprocessing to association rules mining, the mining results in a visual form, based on the probability and stochastic convolution neural network algorithm to generate association rules analysis from the student to study the effect of related elements, namely, the formation of the advantages and disadvantages of the current study effect, help teachers scientific optimization of sports teaching.  Table 2 shows the PE learning effect of a student mined based on the method in this study.

Table 2 only shows part of the data mining results of this student. Support and trust are preset values, which are inversely proportional to the number of mining rules and directly proportional to the mining efficiency. The greater the support is, the less rules are generated and the higher the efficiency of mining association rules is. In order to weigh the number and efficiency of mining association rules for sports learning effect, the support degree is defined between 35%–50%, which can not only guarantee the mining results of learning effect in a short time but also guarantee the number of mining association rules.

The location adaptive probabilistic stochastic convolutional neural network approach will be evaluated using two large scale physical education evaluation validity estimation datasets COCO and MHP. The COCO dataset contains 200,000 images and a total of 250,000 human samples. The images in COCO were taken in an uncontrolled environment, making the human samples challenging. Most images in the COCO dataset are labeled with 17 types of nodes. Use COCO's official public data partition method. The MPII dataset, collected from the YouTube video website, covers the daily life of humans. There are about 25,000 images in the dataset, including 40,000 human body samples, most of which are labeled with 14 types of points.

## 3. Results and Analysis

The effectiveness of the position adaptive probabilistic stochastic convolutional neural network method and the simplified training strategy are evaluated in the COCO verification set. In order to make a fair comparison, the marked human body detection frame provided by COCO dataset was adopted in the experiment, and the input image was not flipped left and right during the test. The effectiveness verification of the position adaptive probabilistic stochastic convolutional neural network method is shown in Table 3.

**Figure 5:**
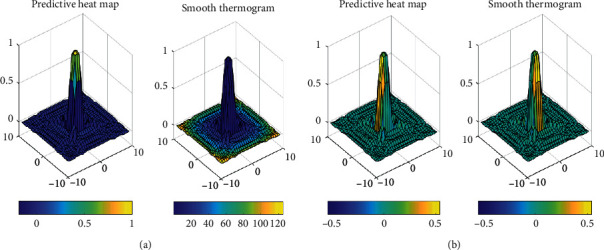
Visualization of thermal map of PE teaching evaluation based on probabilistic stochastic convolution neural network. (a) Sample 1. (b) Sample 2.

Visualization analysis is carried out on the thermal diagram predicted by the model, and the visualization results are shown in Figure 5(a).  Figure 5 is the thermal diagram predicted by the model. It can be seen that there are multiple peaks around the maximum activation value of the thermal diagram predicted by the model because the effectiveness of PE teaching evaluation of probabilistic stochastic convolution neural network is based on the accuracy of the joint coordinate decoding method of Taylor expansion.

**Figure 6:**
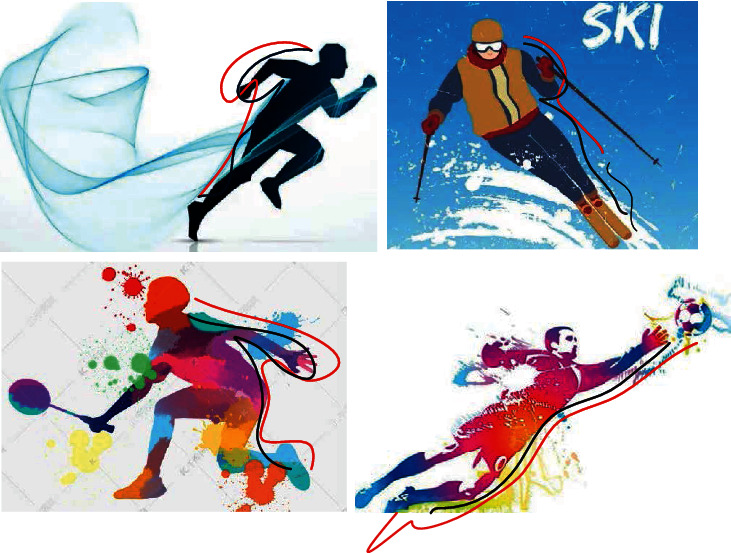
Visualized results on the COCO test set.

In addition to quantitative analysis, Figure 6 also visualizes part of the predicted results of the COCO test set. In the figure, black is the prediction result of probabilistic stochastic convolution neural network method and red is the prediction result of stochastic convolution neural network method. From the comparison results in the figure, it can be seen that the proposed method can still predict the precise human posture under the unrestricted environment and complex background.

**Figure 7:**
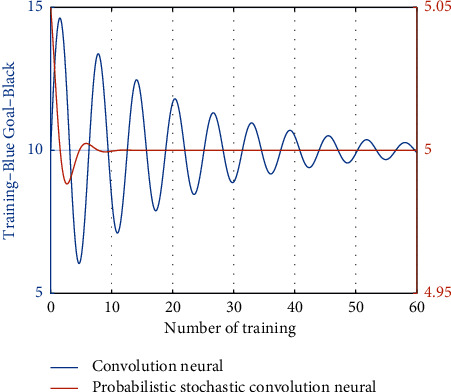
Training results.

**Figure 8:**
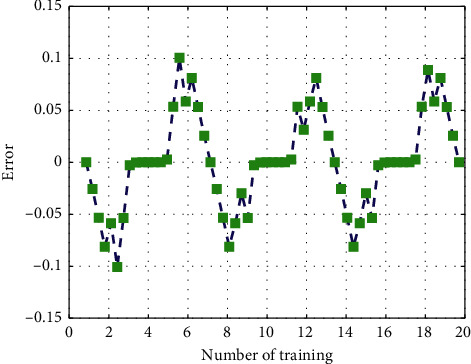
Error curve.

The network training function is shown in Figure 7. After 2000 times of training, the output error of the network is relatively large, and the convergence rate of the network error is very slow. This is because the training function Traingd is a simple gradient descent training function, the training speed is relatively slow and easy to fall into the local minimum situation. The error curve of the network is shown in Figure 8.

According to the above steps, the system conducts simulation training experiments in the MATLAB neural network tool box. The evaluation results of network training and expert evaluation are shown in Table 4. It can be seen from Table 4 that all training samples are close to the expert evaluation results.

In order to verify the evaluation effect of the model, five groups of test data prepared in advance were input into the trained neural network. The simulation results and expert evaluation results are shown in Table 5. The simulation results are close to the evaluation results given by experts.

The comparison between Tables 4 and 5 shows that not only the training and prediction accuracy are completely within the acceptable range but also the error of the test sample is very close to the error of the test sample. Therefore, the student evaluation model based on probabilistic stochastic convolution neural network is a reasonable and feasible evaluation model.

## 4. Conclusion

Probability and stochastic convolution neural network used in physical education teaching evaluation of the effectiveness of the concrete implementation: physical education teaching effectiveness evaluation of every evaluation index of quality evaluation system of probability and stochastic convolution neural network input vector, using the value probability and stochastic vector convolution of the neural network output, through the reasonable design of network structure as well as the training sample. The training samples are input into the network for operation until the system error meets the specified requirements, and the network model is the required comprehensive evaluation model for the effectiveness and quality of physical education evaluation. The results of MATLAB simulation analysis verify that not only the prediction accuracy of training samples is completely within the acceptable range but also the error of test samples is very small, which proves that the teaching quality evaluation model based on probabilistic stochastic convolutional neural network is a reasonable and feasible evaluation model. At the same time, experiments verify that when the neural network uses probabilistic stochastic convolution neural network learning algorithm, the network has the fastest convergence speed and the smallest error. On feature extraction in this study, the extraction of basic skeleton space characteristics of the human body gesture can be based on sports action to achieve a good description, but for human daily or complex sports action which cannot achieve very good expression, the future can continue to study for a more general movement posture feature extraction and match for the human daily and complex movement and identification function.

## Figures and Tables

**Table 1:** Physical education student evaluation index system.

Level indicators	No.	The secondary indicators
Teaching attitude	*X*1	(1) Abide by laws and regulations, without being late, leaving early, missing classes, or adjusting classes frequently
	*X*2	(2) Love students, willing to communicate with students and care about the growth of students
	*X*3	(3) Rigorous academic research, careful lesson preparation and homework correction, and serious and responsible in answering questions and solving doubts, and fully prepared teaching AIDS (courseware)

Teaching content	*X*4	(1) The teaching purpose is clear, the focus is prominent, and the difficulty is appropriate and good at teaching in accordance with their aptitude
	*X*5	(2) The language is accurate, concise, prominent, and skilled use of modern teaching methods to assist teaching, and the effect is obvious
	*X*6	(3) Good at combining theory with practice, pay attention to the development of students' intelligence, and encourage innovation

Teaching method	*X*7	(1) The professional theoretical level is high, the practical skills are strong, and the updated knowledge can reflect the requirements of the times
	*X*8	(2) Can closely follow the outline and contact the actual; the example is appropriate
	*X*9	(3) The classroom atmosphere is enthusiastic; students can actively participate in the whole teaching process

Teaching effect	*X*10	(1) Through teaching, students have mastered the theoretical knowledge required by the course and improved their practical ability
	*X*11	(2) After teaching, students' self-learning ability of related majors or subsequent courses has been improved significantly
	*X*12	(3) Through teaching, students can discover or solve some problems in the major or the course

**Table 2:** Mining association rules for PE learning effect of a student.

Data mining content	Support (%)	Trust (%)
Hobbies: jogging; endurance test score (good)	45.56	68.26
Flexibility training (good)	43.23	90.42
Long-distance running result (good)	35.43	88.76
Physical coordination (excellent); aerobics performance (excellent)	42.38	89.34
Physical dexterity (general) professional badminton competition (general)	40.22	82.06

**Table 3:** Evaluation results of the position adaptive probabilistic stochastic convolution neural network method.

Method	mAP	AP50	AP75	Mapm	Mapl	mAR
HeatMap	60.3	88.2	67.8	58.5	62.8	64.9
Stochastic neural network	60.2	86.1	67.9	59.3	63.4	65.3
Probabilistic stochastic convolutional neural network	63.2	88.7	69.9	61.4	65.8	66.8

**Table 4:** Comparison table between expert evaluation results and probabilistic stochastic convolutional neural network evaluation results.

Test sample number	Expert evaluation value	Expert evaluation results	Evaluation value of neural network	Evaluation results
1	0.92	Good	0.923	Good
2	0.72	Medium	0.723	Medium
3	0.84	Good	0.845	Good
4	0.63	Pass	0.634	Pass
5	0.72	Medium	0.734	Medium
6	0.75	Pass	0.76	Pass
7	0.84	Good	0.856	Good
8	0.65	Pass	0.654	Pass
9	0.67	Pass	0.657	Pass
10	0.73	Medium	0.734	Medium

**Table 5:** Comparison table between simulation evaluation results and expert evaluation results.

Test sample number	Expert evaluation value	Expert evaluation results	Evaluation value of neural network	Evaluation results
1	0.54	Do not pass	0.56	Do not pass
2	0.69	Pass	0.698	Pass
3	0.64	Pass	0.645	Pass
4	0.69	Medium	0.699	Medium
5	0.75	Good	0.77	Good

## Data Availability

The data used to support the findings of this study are available from the corresponding author upon request.

## References

[1] Yan X. D. (2017). Research on the effectiveness of college physical education teaching based on network media. *Education Teaching Forum*.

[2] Wenwen L. (2019). Modeling and simulation of teaching quality in colleges based on BP neural network and training function. *Journal of Intelligent and Fuzzy Systems*.

[3] Cao X., Wang Y., Shi Z. (2021). Research on the maintenance effectiveness evaluation of electronic information equipment. *Journal of Physics: Conference Series*.

[4] Miller L., Sloniewsky M., Gibbons T., Johnston J., Vosler K., Nasir S. (2017). Long-term clinical benefit and cost-effectiveness of an 8-week multimodal knee osteoarthritis management program incorporating intra-articular sodium hyaluronate (Hyalgan&reg;) injections. *Journal of Pain Research*.

[5] Seidu S., Khunti K., Yates T. (2021). The importance of physical activity in management of type 2 diabetes and COVID-19. *Therapeutic Advances in Endocrinology and Metabolism*.

[6] Limin Y., Hong W. (2017). Research on the effectiveness of teaching information application based on new media thinking analysis. *Revista de la Facultad de Ingenieria*.

[7] Sun Y. (2015). Research on development of physical fitness in physical education teaching. *The Open Cybernetics & Systemics Journal*.

[8] Dillenbourg P. (2016). The evolution of research on digital education. *International Journal of Artificial Intelligence in Education*.

[9] Liu Y., Tan F. Q. (2019). Research on the application of the mixed teaching of public physical education in the new media era. *Journal of Shaoguan University*.

[10] Peng L., Zuxing H. E., Department P. E. (2016). Research on the application of the humanistic spirit of sports teachers in the teaching of public physical education. *The Guide of Science & Education*.

[11] Liu S. (2021). Research on the teaching quality evaluation of physical education with intuitionistic fuzzy TOPSIS method. *Journal of Intelligent and Fuzzy Systems*.

[12] Li M. (2017). Research on the influence of developmental education evaluation on college student’s learning effect in physical education elective courses. *Bulletin of Sport Science & Technology*.

[13] Wang J., Ying W. H. (2015). Research on the evaluation system of the balanced development of physical education in China. *International Journal of Simulation: Systems*.

[14] Liu F. (2021). Era of big data is based on the study of physical education teaching mode in MOOC. *Journal of Physics: Conference Series*.

[15] Yan H., Ting Y. (2018). The effectiveness of online citizen evaluation of government performance: a study of the perceptions of local bureaucrats in China. *Public Personnel Management*.

[16] Min J. C. (2017). Research on the influence of humanized physical education model on the physical and mental health of college students. *Journal of Shaanxi Xueqian Normal University*.

[17] Pacchiano D. M., Whalen S. P., Horsley H. L. (2016). Efficacy study of a professional development intervention to strengthen organizational conditions and effective teaching in early education settings. *Society for Research on Educational Effectiveness*.

[18] Wang C. K., Wei G. H. (2017). Theory research on establishing traditional national physical education in PE professional of colleges in guangxi. *Journal of Xichang College(Natural Science Edition)*.

